# *Bcl11b *mutations identified in murine lymphomas increase the proliferation rate of hematopoietic progenitor cells

**DOI:** 10.1186/1471-2407-7-195

**Published:** 2007-10-17

**Authors:** Anneli Karlsson, Amanda Nordigården, Jan-Ingvar Jönsson, Peter Söderkvist

**Affiliations:** 1 Division of Cell Biology, Department of Biomedicine and Surgery, Faculty of Health Sciences, Linköping University, S-581 85 Linköping, Sweden

## Abstract

**Background:**

The telomeric region of mouse chromosome 12 has previously shown frequent allelic loss in murine lymphoma. The *Bcl11b *gene has been identified and suggested as a candidate tumor suppressor gene within this region. In this study, we aimed to elucidate whether *Bcl11b *is mutated in lymphomas with allelic loss, and whether the mutations we detected conferred any effect on cell proliferation and apoptosis.

**Methods:**

Mouse lymphomas induced by 1,3-butadiene or 2',3'-dideoxycytidine were analysed for mutations in the *Bcl11b *gene using single strand conformation analysis and direct DNA sequencing. Effects on cell proliferation by the detected mutations were studied by expressing wild-type and mutant Bcl11b in the cytokine-dependent hematopoietic progenitor cell line FDC-P1, lacking endogenous Bcl11b expression.

**Results:**

Missense and frameshift (FS) mutations were identified in 7 of 47 tumors (15%). Interestingly, all mutations were found between amino acids 778–844 which encode the three C-terminal DNA-binding zinc fingers. In FDC-P1 cells, wild-type Bcl11b suppressed cell proliferation, whereas the mutated versions (S778N, K828T, Y844C and FS823) enhanced proliferation several-fold.

**Conclusion:**

The genetic alterations detected in this study suggest that the three C-terminal zinc fingers of Bcl11b are important for the DNA-binding. Cell proliferation was suppressed by overexpression of wild-type Bcl11b but enhanced by mutant Bcl11b, indicating that these mutations may be an important contributing factor to lymphomagenesis in a subset of tumors.

## Background

The *BCL11B*/*Rit1*/*CTIP2 *gene was first identified in human chromosome 14q32.2 [1], as a homologue to *BCL11A*/*CTIP1*, which is known to be involved in translocations in human leukemia [2,3]. In the immune system, BCL11B is expressed exclusively in the T cells [4] and is involved in both translocations [5] and inversions [6] in human T-cell acute lymphoblastic leukemia (T-ALL). Also, deletions in the *Bcl11b *gene have been detected in radiation-induced lymphoma in mice that is caused by V(D)J recombinase activity [7]. *Bcl11b*^-/- ^knockout mice show a block at the immature stage of T-cell differentiation, partly due to lack of pre-T-cell receptor (TCR) expression on the cell surface [4]. Introducing functional TCRβ and TCRαβ-chains into *Bcl11b*^-/- ^mice does not release this differentiation block, suggesting another signaling mechanism required for differentiation of T cells [8].

BCL11B contains six DNA-binding zinc-finger structures as well as a proline-rich domain and an acidic domain that may possibly transactivate target genes [1]. Bcl11b has been shown to be a strong transcriptional repressor in vitro [9], and other groups have shown that Bcl11b interacts with the histone deacetylase SIRT1 within a larger protein complex in mammalian cells [10], and with the nucleosome remodeling and deacetylase (NuRD) protein complex in T lymphocytes [11]. Both SIRT1 and NuRD bind to and deacetylate p53, thereby repressing p53-mediated transactivation [12,13]. Recently, Cismasiu *et. al*. [14] reported that Bcl11b initiates IL-2 transcription in CD4+ T cells, contradictory to the previous report on Bcl11b being a transcriptional repressor [9]. Bcl11b has also been assigned anti-apoptotic properties, since knock-down of Bcl11b expression with RNA interference induced apoptosis in T-cell lines [15,16].

Murine *Bcl11b *shows 88% identity to the human *BCL11B *at nucleotide level. Studies on chemically induced T-cell lymphoma in mice have previously shown frequent allelic loss in the telomeric region of chromosome 12 [17], which is syntenic to human 14q32.2 and the location of the *Bcl11b *gene. Another study revealed frequent mutations in *Bcl11b *in radiation-induced lymphoma in mice [18], supporting a role of Bcl11b in regulation of cell growth. The present study identifies tumor specific point mutations and microdeletions in chemically induced mouse lymphoma. The mutations were functionally analysed by overexpression in the hematopoietic progenitor cell line FDC-P1. Interestingly, wild-type Bcl11b was able to suppress cell proliferation, whereas tumor specific point mutations and frameshift mutations in *Bcl11b *enhanced proliferation.

## Methods

### Materials

Sixteen 2',3'-dideoxycytidine-induced lymphoma in C57Bl/6 × C3H/HeJ F_1 _(B6C3F1) mice (DLF) and thirty-one 1,3-butadiene-induced lymphoma in B6C3 F1 mice (BLF) were analyzed for mutations in the *Bcl11b *gene. The tumors were kindly provided by R. Wiseman (National Institute of Environmental Health Sciences, Research Triangle Park, NC) and were induced by gavage of 2',3'-dideoxycytidine [19] or by inhalation of 1,3-butadiene [20]. In total 47 lymphomas (31 BLF and 16 DLF) were collected, all of T-cell origin [19,20]. DNA was purified as previously described [17].

### Mutation analysis

Mutations were detected with Single Strand Conformation Analysis (SSCA) and direct sequencing. The *Bcl11b *gene was PCR-amplified for 35 cycles in the 47 tumor samples and in normal spleen from B6C3F1 mice. Primers were designed to cover the complete coding sequence of *Bcl11b *including exon/intron borders (Table 1). The amplified DNA was radioactively labeled with [α-^32^P]dATP (Amersham Pharmacia Biotech, Buckinghamshire, UK) for 10 cycles, denatured and applied to a non-denaturing 6% polyacrylamide gel containing 10% glycerol and to an 0.5 × MDE™-gel (FMC BioProducts, Rockland, ME). Fragments with altered electrophoretic mobility were excised from the gel, eluted in water and reamplified for 35 cycles. The PCR product was purified with ExoSAP-IT^® ^(Amersham Biosciences, Uppsala, Sweden), labeled with DYEnamic ET Dye Terminator Cycle Sequencing Kit for MegaBACE DNA Analysis Systems™ (Amersham Biosciences), following the manufacturer's instructions, and applied to MegaBACE DNA Analysis Systems™ (Amersham Biosciences). Repeated mutation analysis from original DNA was performed on tumors to confirm mutations.

**Table 1:** Primers used for mutation analysis, sequencing, cloning and mutagenesis of mouse *Bcl11b*.

Fragment	Direction	Sequence (5'->3')	Size of PCR product (bp)	Annealing temp. (°C)/Cycles
ex1	F	CGC ATC TGT GCA GCT TTC	226	56/35
	R	CCG GCT GCA GAG AAA CTT		
ex2a	F	TGT CTT CTC TGC CCC TTC C	264	59/35
	R	CTT GTC CAG GAC CTT GTC GT		
ex2b	F	ACC TGT GGC CAG TGT CAG AT	300	59/35
	R	TAG GGA GGC AGC TTC AGC A		
ex3	F	GGC ACC ACT GAC CAG TCT TT	377	61/35
	R	CTT TTT GAG TGG GGG ATG G		
zfd1	F	TGC TCA CAC TCT GCC TTT CTT	291	59/35
	R	GCA GCA GGT TGA AAG GAT TG		
ex4a	F	TGA ATT TCC TGG GGG ACA G	283	60/35
	R	CTG TTG CCG GCC AGT TCT		
prd	F	TCT CCT GCC ATG GAC TTC TC	329	62/35
	R	TGG CAC TTG TAG GGC TTC TC		
zfd2	F	AGT CCC AAG TCC CCG TTC	299	60/35
	R	CAG CTG AGA GCC CGT CGT		
acd	F	ATG AAG ACG CAC ATG CAC AA	289	64/35
	R	CAC GCC ACC TCC GTT CTC		
ex4b	F	AGC ATG GAC TCG GAG CTG	300	62/35
	R	AGC TGG AAA GGG CTC TGC		
ex4c	F	GTG ATG CCG GAG CTG TTG	291	59/35
	R	CCA GGA ATG GGT CCT TCA		
ex4d	F	GTG TAC TCG CAG TGG CTC GT	294	60/35
	R	GAC CTT GCC GCA GTA CTC AC		
zfd3	F	ACA CCT CAC CTG GGT GGT C	274	59/35
	R	TCA TTA GTC AGC AAG TGT TCA CC		
				
cloning	F	TTT GAA TTC ATG TCC CGC CGC AAA C	2459	65/35
	R	TTT GCG GCC GCT TAG CTC CTC TCA GCC TGC TC		
				
S778N	F	CTG GGA GGC CGA ACT CCA AGG AGG GC		65/12
	R	GCC CTC CTT GGA GTT CGG CCT CCC AG		
K828T	F	GCA GAG CAG CAC GCT CAC GCG CCA C		65/12
	R	GTG GCG CGT GAG CGT GCT GCT CTG C		
Y844C	F	GCA AGG AGG TGT GCC GCT GCG ACA TC		65/12
	R	GAT GTC GCA GCG GCA CAC CTC CTT GC		
FS823	F	CGA GCT GTG CAA CTA CGC GCT AAG AGC AGC AAG CTC ACG CG		65/18
	R	CGC GTG AGC TTG CTG CTC TTA GCG CGT AGT TGC ACA GCT CG		

### Transfection of FDC-P1 and generation of Bcl11b expressing clones

cDNA corresponding to wild-type *Bcl11b *was PCR-amplified using the primers in Table 1 and ligated into the expression vector pCI-neo (Promega, Madison, WI). S778N, K828T, Y844C and FS823 were introduced into the plasmid using the protocol of Quikchange (Stratagene, La Jolla, CA) and the primers presented in table 1. Wild-type and mutant plasmids were then individually transfected into the IL-3-dependent hematopoietic progenitor cell line FDC-P1 by electroporation (260 V, 950 μF). Forty-eight hours post-transfection, 1,000 cells/well were seeded on a 96-well plate in Iscove's modified Dulbecco's medium (IMDM) supplemented with IL-3, 10 % fetal calf serum and 1 mg G418/ml (Gibco) to select for transfected cells. G418-resistant clones were picked, expanded, and tested for expression of Bcl11b by RT-PCR and Western blot. RNA was isolated from the cells using Trizol^® ^reagent (Invitrogen, Carlsbad, CA) according to the manufacturer's instructions. RT-PCR was performed by an initial denaturation of 5 μg of RNA and 0.25 μg random primers (Invitrogen) in 11.5 μl volume for 10 minutes at 70°C. To this sample, 200 U RT Superscript (Invitrogen) and 13 U RNAguard (Amersham Biosciences) was added in a total volume of 20 μl of 1 × buffer (Invitrogen), 0.5 mM dNTP, 0.01 M dithiothreitol (final concentrations), prior to incubation at 42°C for 50 minutes and then 70°C for 15 minutes. The cDNA was used for PCR with primers ex2bF and zfd1R (Table 1). For Western blot analysis, cells were lysed in RIPA buffer and the protein fraction was separated on a 4–12% gradient PAGE gel (Invitrogen) and blotted to a PVDF membrane. Bcl11b expression was detected with antibodies against human BCL11B (Bethyl Laboratories Inc., Montgomery TX), showing partial cross-reactivity to mouse Bcl11b.

### FACS analysis of cell survival

FDC-P1 cells were washed twice in PBS and twice in PBS with 10% FCS and seeded in a 24 well plate (1 ml/well) at the initial density of 5 × 10^5 ^cells/ml and in the absence of IL-3. After 24, 48 and 72 hours of incubation at 37°C and 5% CO_2_, cells were washed twice in PBS and the proportion of dead cells measured by flow cytometry on a FACS Calibur flow cytometer (Becton Dickinson, San Jose, CA) after staining cells in 0.8 μg/ml of propidium iodide (final concentration).

### Determination of cell proliferation

Cells were washed twice in PBS and twice in PBS with 10% FCS, and then cultured at 1 × 10^4^/well in 96-well flat-bottom microtiter plates. Cultures were maintained for 72 hours at 37°C and 5% CO_2 _in IMDM supplemented with 10% FCS and stem cell factor (SCF; PeproTech EC Ltd., London, UK) at 100 ng/ml. At the end of the experiment, cultures were labeled for 6 hours with 0.5 μCi/well [^3^H]-thymidine (Amersham Biosciences), harvested, and counted in a β-scintillation counter.

### CFSE staining

CFSE, 5-(and-6)-carboxyfluorescein diacetate succinimidyl ester (Molecular Probes, Eugene, OR), stock solution (5 mM in DMSO) was diluted 1:1000 in IMDM (no FCS). Cells were washed in PBS and then resuspended in CFSE/IMDM for 8 minutes at room temperature in the dark. Labeling was quenched by adding the same volume of FCS, inverting the tube for 2–3 seconds followed by centrifugation. Cells were washed four times in IMDM with FCS (10 %), and then cultured in 100 ng/ml SCF. After 72 hours, cells were washed once in PBS and analyzed for CFSE staining on a FACSCalibur cytometer (Becton Dickinson) on the FL-1 channel using log amplification.

## Results

Forty-seven chemically induced lymphomas were analysed for mutations in the *Bcl11b *gene by SSCA and MegaBACE sequencing. In total, seven of 47 tumors (15%) revealed mutations, of which six were missense mutations and one was a frameshift mutation. BLF17 displayed a 7 bp-deletion and a 3 bp-insertion, causing a frameshift mutation in codon 823 and a premature truncation at codon 832. The truncation results in deletion of two C-terminal zinc fingers of the Bcl11b protein. Interestingly, four samples (BLF3, BLF4, BLF15 and DLF16) displayed A>C mutations in base 2483, indicating a hotspot site for mutations in 1,3-butadiene and 2',3'-dideoxycytidine induced lymphomas. This mutation results in an exchange of the basic amino acid lysine to the neutral threonine in codon 828. This codon is located in the fifth zinc finger of the Bcl11b protein, which may be important for DNA binding to target genes. DLF1 showed an A>G mutation at base 2531 with a subsequent tyrosine to cysteine substitution in codon 844. The functional consequence of this amino acid shift is unknown, but the shift occurs only two amino acids away from one of the zinc-binding cysteines in the sixth zinc finger and may affect the structure of the zinc fingers and thus the DNA-binding. BLF22 revealed a G>A mutation in base 2333, resulting in a serine to asparagine substitution in codon 778. This amino acid is not included in any of the zinc fingers, but the change from a serine could possibly result in loss of a phosphorylation site, which would interfere with the function of the protein.

**Figure 1:**
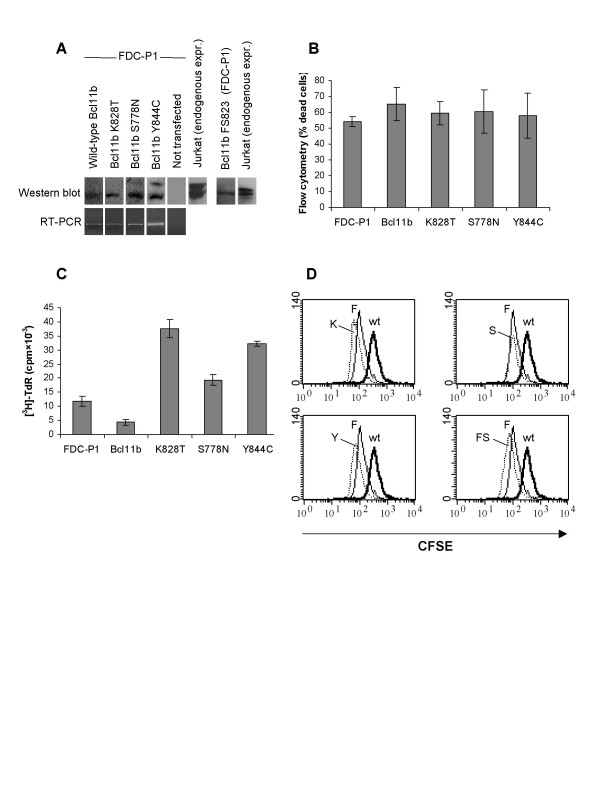
**Overexpression of *Bcl11b *inhibits proliferation of the hematopoietic progenitor cell line FDC-P1 but has no effect on apoptosis**. (**A**) Expression of Bcl11b in FDC-P1 and cells transfected with the mutated versions K828T, S778N, Y844C and FS823 was detected by Western blot and RT-PCR. In the Western blot analysis, the human cell line Jurkat was included as a positive control. The two bands correspond to different isoforms of Bcl11b. (**B**) Cell viability was determined by staining with propidium iodide followed by flow cytometric analysis. Data shown are the mean ± SD (n = 6). (**C**) Proliferation of non-transfected FDC-P1 cells and FDC-P1 cells transfected with wild-type Bcl11b or the mutated versions (K828T, S778N or Y844C) was measured by [^3^H]-TdR incorporation (cpm × 10^-3^) after 72 hours of SCF-stimulation. Results are presented as mean ± SD (n = 3). FDCP1: 11.8 ± 1.7; wild-type Bcl11b: 4.4 ± 1.1; K828T: 37.5 ± 3.3; S778N: 19.3 ± 1.9; Y844C: 32.4 ± 0.9. (**D**) Cells were stained with CFSE and cultured for 72 hours in SCF, after which CFSE fluorescence was determined by flow cytometry. Histograms for the four different mutants K828T, S778N, Y844C and FS823 (K, S, Y and FS, respectively) are shown. CFSE fluorescence declines (shift to the left on the X-axis) as cell numbers increase. In each histogram, data from non-transfected FDC-P1 cells (F) and cells transfected with wild-type *Bcl11b *(wt) are overlaid for a comparison. Results are from one representative experiment of three performed.

To elucidate if any of these mutations influence cell function, FDC-P1 cells were transfected with either wild-type *Bcl11b *or any of three point mutated versions of *Bcl11b *(S778N, K828T and Y844C). We chose FDC-P1 cells for these experiments due to lack of endogenous Bcl11b expression and because of the wide use of this cell line to study proliferation and apoptosis in hematopoietic cells. FDC-P1 cells are dependent on IL-3 for continuous growth but are responsive to other cytokines such as SCF by slower cell cycle kinetics and inhibition of apoptosis. In addition, SCF can synergize with other stimuli to enhance proliferation [21]. Multiple clones were generated for either wild-type *Bcl11b *or the different mutants after selection in G418. For subsequent analyses, however, only few clones were chosen for each case. Expression was first detected by RT-PCR (Figure 1A). To quantify the amount of Bcl11b expression, Western blots were performed using an antibody against human BCL11B which displays some cross-reactivity to mouse Bcl11b (Figure 1A). The clones were initially grown in the absence of cytokines to analyze the ability to sustain survival as measured by propidium iodide staining followed by flow cytometry. However, none of the clones were able to support survival (Figure 1B). In addition, Bcl11b was analyzed for synergistic effects in combination with SCF by [^3^H]-TdR incorporation. Interestingly, FDC-P1 cells transfected with the non-mutated *Bcl11b *gene showed a two-three-fold suppression of proliferation compared to non-transfected cells (Figure 1C). In contrast, K828T, S778N and Y844C were able to enhance SCF-induced proliferation two-three-fold compared to non-transfected FDC-P1 (Figure 1C).

To confirm that the wild-type Bcl11b gene inhibits and that the mutants enhance proliferation, we measured cell cycle progression of FDCP-1 cells by staining them with the fluorescent marker CFSE. In this assay, we also tested whether the truncated mutant FS823 had effects on cell proliferation. After culturing the cells for 72 hours in the presence of SCF and CFSE, we performed flow cytometric analysis. This is a widely used method to assess proliferation rate, since the intensity of the CFSE stain gradually decreases as cells divide. As seen in Figure 1D, the intensity of CFSE is diminished in cells transfected with mutant K828T and Y844C, whereas the S778N mutant did not differ compared to untransfected cells. This is in agreement with the results from the [3H]-TdR incorporation (Figure 1C), where the S778N mutant did not increase proliferation much compared to non-transfected cells. Three individual clones carrying the FS823 mutation were also included in this proliferation assay, where they displayed a similar decrease as K828T and Y844C (only data from one clone is shown in Figure 1D). Importantly, cells transfected with wild-type Bcl11b displayed higher CFSE intensity than untransfected FDCP-1, indicating that these cells have passed through fewer cell cycles.

**Figure 2:**
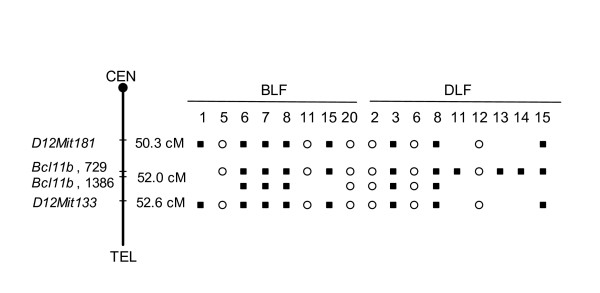
**Allelic loss of the C3H allele or the B6 allele on chromosome 12 in BLF and DLF**. Two polymorphic sites (729A>G and 1386G>A) were found in *Bcl11b*, confirming previously detected allelic loss in *D12Mit181 *and *D12Mit133 *[17]. Markers analyzed are shown on the left side of the chromosome and the distance of the marker from the centromere is presented on the right side of the chromosome. Black square indicates loss of the C3H allele; white circle indicates loss of the B6 allele; absence of circle or square indicates retention of heterozygosity. CEN, centromere; TEL, telomere.

Apart from identifying mutations in the *Bcl11b *gene, sequencing also revealed two polymorphic sites (729A>G and 1386G>A) in the C3H/HeJ strain compared to the C57Bl/6J strain. Neither of the polymorphisms results in amino acid change and is therefore not likely to affect the function. These polymorphisms enabled us to confirm previously detected allelic loss in chromosome 12 in 16 out of 47 samples [17] (Figure 2). None of the *Bcl11b *mutated samples coincided with the samples showing allelic loss, contradictory to previous reports on mutations in *Bcl11b *[18].

## Discussion

The region on mouse chromosome 12 where *Bcl11b *is located has previously shown frequent allelic loss in murine lymphomas [17]. The role of Bcl11b in lymphomagenesis is also indicated by studies showing *Bcl11b *mutations in radiation-induced mouse lymphomas [18] as well as inversions and translocations involving *Bcl11b *in human T-ALL [5,6]. This is further supported in the present study, where 15% (7/47) of chemically induced murine lymphoma (13% of DLF and 16% of BLF) display somatic mutations in *Bcl11b*. Interestingly, all mutations are located between codon 778 and 844, with a hotspot at codon 828 where four samples show the same mutation (Table 2). This region of Bcl11b contains three of the six zinc finger structures, which concomittant with studies on other zinc-finger containing transcription factors are likely to be important for the DNA-binding function of the protein. The frameshift mutation was detected in the same region, deleting the two C-terminal zinc fingers.

**Table 2:** Summary of genetic alterations of the *Bcl11b *gene in BLF and DLF.

Samples	Mutations in *Bcl11b *(n = 47)		Mutations in *p53*^a^
		
	Nucleotide	Amino acid	
DLF			
1	2531A>G	Y844C	
16	2483A>C	K828T	
BLF			
3	2483A>C	K828T	
4	2483A>C	K828T	
7			N236S
8			C173F
9			Frameshift
10			H176L
12			Frameshift
15	2483A>C	K828T	
17	2467TGCGCGC>CTA	Frameshift in codon 823	
22	2333G>A	S778N	
25			R153G

^a^Mutations of *p53 *have previously been analyzed and reported [26].

To test whether the mutations detected in this study had any effect on cell growth, FDC-P1 cells lacking endogenous expression of Bcl11b were transfected with either wild-type *Bcl11b *or any of the four mutants S778N, K828T, Y844C or FS823. FDC-P1 cells overexpressing wild-type Bcl11b showed reduced proliferation rate with more than 50% compared to non-transfected cells. Overexpression of the K828T, Y844C and FS823 mutated variants of Bcl11b resulted in increased proliferation both with thymidine incorporation (Figure 1C) and with CFSE staining (Figure 1D). Interestingly, the K828T mutation that was detected in four tumor samples seemed to increase proliferation rate most efficiently among the three different missense mutants. In addition, the frameshift mutant also increased cell growth to the same extent as K828T. These data demonstrate that the mutations identified in the present study are functional and affect cell proliferation.

Allelic loss has previously been analyzed in the tumors of this study [17] and the LOH was here confirmed by two intragenic single nucleotide polymorphisms in *Bcl11b *identified during the present mutation analysis on *Bcl11b *(Table 2). Wakabayashi *et *al [18] has previously detected point mutations and allelic loss in radiation-induced lymphoma in agreement with a two-hit hypothesis. However, none of the samples in this study with mutations in *Bcl11b *showed simultaneous allelic loss (Figure 2; Table 2). Kamimura *et. al*. [22] suggests that Bcl11b may be haploinsufficient for suppression of tumors, since *Bcl11b*^+/-^*p53*^+/- ^mice generates more spontaneous tumors than do *Bcl11b*^+/+^*p53*^+/- ^mice. The phenomenon of haploinsufficiency, where one single copy of the gene may be incapable of providing sufficient protein in order to maintain normal cell function, may explain the findings of our study. It has previously been demonstrated for other tumor suppressors as well, e.g. PTEN [23] and Smad4 [24].

Our study adds novel information on the growth enhancing effect of mutated Bcl11b. Our results are also in agreement with the report by Wakabayashi et al. [18] demonstrating the ability of non-mutated Bcl11b to suppress tumor growth when introduced into HeLa cells. These authors identified similar but not identical mutations in the *Bcl11b *gene in radiation-induced mouse lymphomas. Deletions were also detected in the radiation-induced lymphomas, however, these were later found also in normal thymocytes at an equal frequency [7].

The mechanisms by which Bcl11b execute growth suppression is still unknown. However, a previous report has demonstrated that Bcl11b is able to repress transcription from a consensus response element [9], supporting our notion of a suppressive role for Bcl11b on cell cycle. The same group has recently proved that Bcl11b directly activates IL-2 expression in CD4^+ ^T lymphocytes [14]. However, it is possible that the protein may have both activating and repressing functions depending on which other proteins it associates with and in which cell type it is expressed. This has been described for other zinc finger proteins that are important for T cell proliferation and differentiation, e.g. Ikaros, which represses transcription by binding to the NuRD complex, but may also potentiate gene expression by binding to other proteins [25].

In the study on radiation-induced mouse lymphomas, mutations in *Bcl11b *were found to be mutually exclusive with *p53 *mutations, suggesting a common pathway for tumor formation [18]. Of the 47 tumors analyzed in this study, 13 revealed mutations in either *Bcl11b *or *p53 *[26] (Table 2), but no tumor carried mutations in both genes, supporting mutational complementarity. Furthermore, Bcl11b has been shown to interact with the histone deacetylase protein complex designated NuRD [11], which reduces the levels of acetylated p53, repressing p53 dependent transcription and thereby modulating p53-mediated cell growth arrest and apoptosis [13]. Likewise, the interaction between Bcl11b and another histone deacetylase, SIRT1 [10], also leads to transcriptional repression of p53 [12]. However, due to the effects on proliferation detected in the present study it is possible that Bcl11b may act by an alternative mechanism.

## Conclusion

The genetic alterations detected in this study suggest that the three C-terminal zinc fingers of Bcl11b are important for the DNA-binding. Cell proliferation was suppressed by overexpression of wild-type Bcl11b but enhanced by overexpression of mutant Bcl11b, suggesting that these mutations may be an important contributing factor to lymphomagenesis in a subset of tumors. However, future studies are necessary to elucidate the exact function of Bcl11b.

## Competing interests

The author(s) declare that they have no competing interests.

## Authors' contributions

AK carried out the mutation analysis, preparation of constructs and transfected FDC-P1 cells, FACS analysis, and drafted the manuscript. AN prepared constructs and transfected FDC-P1 cells. JIJ performed FACS analysis and cell proliferation assays. PS conceived of the study and participated in its design. All authors interpreted and discussed the results, and contributed to the final manuscript.

## Pre-publication history

The pre-publication history for this paper can be accessed here:


